# Assessment of the Bacterial Pollution and Detection of Antibiotic Resistance Genes in Benin: Case of the Hydrographic Channel Complex Cotonou-Nokoué Lake

**DOI:** 10.1155/2021/6664816

**Published:** 2021-07-03

**Authors:** Oloufemi Daniel Ichola, Victorien Tamegnon Dougnon, Charles Hornel Koudokpon, Alidehou Jerrold Agbankpe, Esther Deguenon, Aime Cezaire Ayena, Henri Houenoukpo Soclo

**Affiliations:** ^1^Research and Training Laboratory in Applied Chemistry, Polytechnic School of Abomey-Calavi, University of Abomey-Calavi, Godomey, Benin; ^2^Research Unit in Applied Microbiology and Pharmacology of Natural Substances, Polytechnic School of Abomey-Calavi, University of Abomey-Calavi, Godomey, Benin

## Abstract

The study aims to document the level of contamination of the aquatic ecosystem of the Cotonou-Lake Nokoué canal hydrographic complex by multidrug-resistant bacteria and their resistance genes. For this purpose, water samples were taken from several points of the complex and from the sediments at the depth of the lake. Samples of several species of freshly caught fish products from the lake were also collected. Bacteriological analyses were carried out according to the AFNOR standard (NF U: 47–100). The identification of the different bacterial species isolated was then carried out using the API 20E gallery and specific biochemical tests. The antibiogram of the strains was performed according to the recommendations of the EUCAST. Molecular characterization of the identified strains was carried out by searching for resistance and virulence genes. The results obtained revealed the presence of several bacterial species in water samples and in sediment and intestine samples of fishery products with a predominance of Gram-negative bacilli. The resistance profile of Gram-negative bacilli showed a total resistance to metronidazole (100%). 23% of the strains were also resistant to ciprofloxacin, 41% to amoxicillin, and 60% to aztreonam. Of the Gram-positive cocci identified, 66% was resistant to vancomycin, 7.5% to ciprofloxacin, 71% to erythromycin, and 22% to tetracycline. Regarding the genes sought, *bla*_TEM_ (46%), *bla*_SHV_ (24%), and *bla*_CTX-M-15_ (31%) were present in the genome of Gram-negative bacilli as resistance genes and *fimH* (41%) as virulence gene. As for Gram-positive cocci, the *van B* gene was completely absent. The *van A* was present at 6.25% in *Staphylococcus aureus* and *mecA* at 21.88 and 33.33%, respectively, in *Staphylococcus aureus* and coagulase-negative staphylococci strains. The high resistance of isolated bacterial strains is a matter of concern and calls for a rational use of antibiotics in order to avoid the transmission of antibiotic resistance from the environment to humans.

## 1. Introduction

Antimicrobial resistance has become a public health issue that affects both the environment and public health [1]. For several decades, the world's population has been confronted with serious problems related to water security, which, in order to be useful, must meet certain quality criteria [2]. Water, an important molecule in all sectors, whether in human, animal, or plant health, is vital for a normal life [3]. While certain elements are essential for human health, their accumulation in waters, namely, rivers or lakes, can pose a threat to the ecology and human health [4–6]. Several countries in the world, especially those in the Third World, face serious water quality problems [7, 8]. Pollution by microorganisms in rivers and lakes is a major concern for the protection of watershed water quality and public health management [9]. Self-medication has contributed heavily to the selection of antibiotic-resistant bacteria [10]. Bacterial multidrug resistance to antibiotics remains a major public health problem worldwide, in both developed and developing countries. It causes approximately 700,000 deaths each year from antibiotic-resistant bacterial infections [11]. The aquatic environment is very often the final receptacle of various sources of contaminants from industrial, agricultural, hospital, and urban waste discharges [12]. There is a growing interest in exploring the occurrence of antibiotic resistance genes in the environment and the factors that contribute to their emergence. Aquatic ecosystems provide an ideal setting for the acquisition and spread of resistance genes due to the continuous pollution by antimicrobial compounds from anthropogenic activities [13]. Pollution of rivers by multidrug-resistant bacteria has become a problem that cannot be ignored and raises many questions [12]. A few studies in Benin have focused on microbial contamination of water bodies, but very few have provided information on the resistance genes carried by the bacterial strains isolated in their genome. In order to better understand this current public health problem, the present study therefore sets out to document the level of bacterial contamination of the aquatic ecosystem of the Cotonou-Lake Nokoué channel hydrographic complex and to shed light on a few resistance and virulence genes carried by these bacteria in their genome.

## 2. Materials and Methods

### 2.1. Study Area and Sample Collection

**Figure 1:**
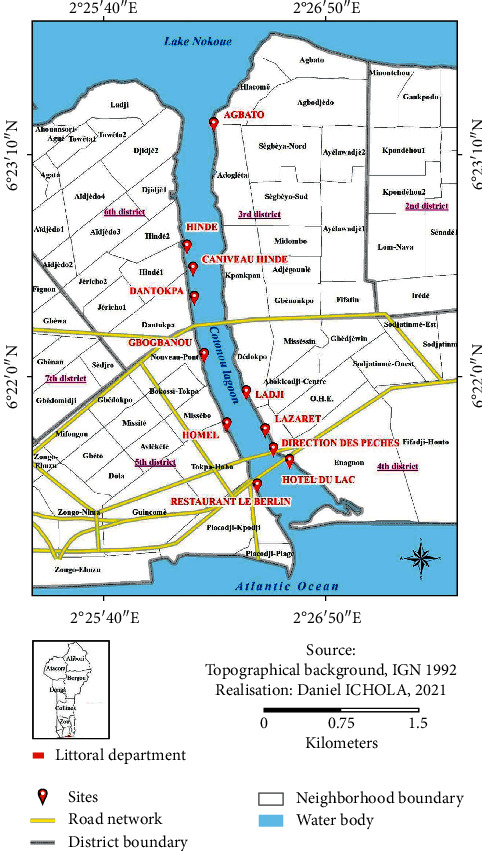
Sample map collection on Lake Nokoué.

The study was carried out in southern Benin between 6°25' N and 7°30' N and covers an area of 17109 km^2^. The climate is subequatorial, characterized by a bimodal rainfall regime with two rainy seasons alternating with two dry seasons. The average annual temperature is 28°C, and air humidity varies between 69% and 97% [14]. The channel hydrographic complex of Cotonou-Lake Nokoué was the actual study area. The samples collected included water and sediment samples from eleven different points in the lake. Also, samples of freshly caught fish products from the lake were included in the sampling.  Figure 1 shows the sampling map.

**Figure 2:**
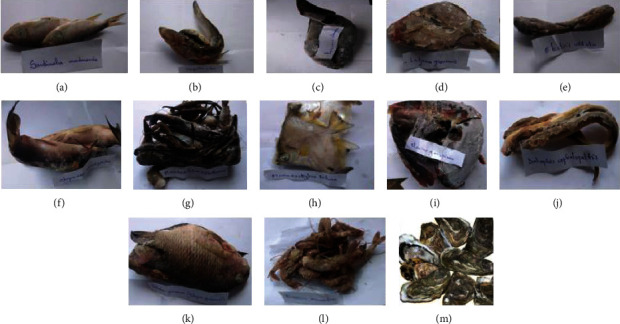
Different species of fishery products sample. (a) *Sardinella madarensis*. (b) *Hepsetus odoe*. (c) *Parachanna obscura*. (d) *Lutjanus groeensis*. (e) *Eleotris vittata.* (f) *Chrysichthys nigrodigitatus*. (g) *Macrobrachium vollenhovenii.* (h) *Monodactylus sebae*. (i) *Clarias gariepinus*. (j) *Dalophis boulengeri.* (k) *Tilapia guineensis*. (l) *Penaeus monodon*. (m) *Crassostrea tulipa*.

A total of 20 waters samples, 10 lake bottom sediment samples, and 13 different species of fish products were collected.  Figure 2 shows the different species of fish products from Lake Nokoué taken during the collection of samples.

### 2.2. Bacteriological Analysis of Samples

Bacteriological analyses were carried out according to the AFNOR standard (NF U: 47–100). 2 times 150 ml of the water samples volume underwent membrane filtration with 0.22 *µ*m diameter membranes. The both filtration membranes were then recovered on eosin methylene blue (EMB) and Chapman agar plates, respectively. Then, the plates were incubated at 37°C for 24 hours.

Each species of fish collected was emptied from the intestines, and 25 g of intestines of each sample was enriched in 225 ml buffered peptone water (BPW) and incubated for 18 hours at 37°C. The sediment samples were also enriched with 25 g introduced into 225 ml of BPW for 24 hours at 37°C. All broths were inoculated by streak on Chapman and eosin methylene blue agar plates for bacterial isolation. These plates were incubated at 37°C for 24 hours. Two or three characteristic colonies obtained on the both culture media used were selected. These colonies were streaked on Mueller-Hinton agar plates for purification and then Gram staining, biochemical tests (catalase and oxidase), seeding of the API 20E gallery (for only Gram-negative bacilli), and free staphylocoagulase and DNAse tests (for only Gram-positive cocci).

### 2.3. Antimicrobial Susceptibility Testing

All identified bacteria were subjected to antimicrobial susceptibility testing on Müller-Hinton agar plates using the Kirby–Bauer disc diffusion method to the following antibiotics: ciprofloxacin (5 *µ*g), erythromycin (15 *µ*g), tetracycline (30 *µ*g), vancomycin (30 *µ*g), metronidazole (5 *µ*g), amoxicillin (25 *µ*g), and aztreonam (30 *µ*g). Measured inhibition zone diameters were interpreted according to EUCAST guidelines [15]. The reference strains *Escherichia coli* ATCC 25922 and *Staphylococcus aureus* ATCC 25923 were used as a control.

### 2.4. Detection of Resistance and Virulence Genes

#### 2.4.1. Extraction of DNA from Isolated Strains

The DNA of the *Salmonella* strains was obtained using the blue Qiagen extraction kit. The tubes containing the DNA were stored at 4°C for the amplification phase. The DNA tubes remaining after handling were stored at −20°C for optimal conservation.

#### 2.4.2. Performance of the PCR

The reaction mixture consisted of 7.5 *μ*L of water, 12.5 *μ*L of the 2x PCR Master Mix reagent, 2 *μ*L of each primer pair, and 3 *μ*L of bacterial DNA. Amplification of the *bla*_TEM_, *bla*_SHV_, and *bla*_CTX-M-15_ genes was performed under reaction conditions involving initial denaturation at 93°C for 4 minutes, followed by 32 cycles at 93°C for 30 seconds, 55°C for 30 seconds, and 72°C for 40 seconds. A final elongation of 4 minutes at 72°C was performed. Amplification of the *qnrA* gene was performed under reaction conditions involving initial denaturation at 95°C for 10 minutes, followed by 40 cycles at 95°C for 30 seconds, 51°C for 30 seconds, and 72°C for 15 seconds. A final elongation of 5 minutes at 72°C was performed. Amplification of the IMP, KPC, GES, NDM, VIM, OXA-48, OXA-23, and DHA genes was performed under conditions involving initial denaturation at 95°C for 5 minutes, followed by 35 cycles at 95°C for 20 seconds, 49°C for 45 seconds, and 72°C for 30 seconds. A final elongation of 5 minutes at 72°C was performed. Amplification of the *fimH* gene was performed under conditions involving initial denaturation at 94°C for 2 minutes, followed by 40 cycles of denaturation at 94°C for 40 seconds, hybridization at 50°C for 1 minute, and initial elongation at 72°C for 1 minute. A final elongation was initiated at 72°C for 5 minutes. For the PCR of Gram-positive cocci, the *mecA*, *van A,* and *van B* genes were searched. Amplification was performed under conditions involving initial denaturation at 94°C for 4 minutes, followed by 30 cycles at 94°C for 1 minute, at 50°C for 1 minute, and at 72°C for 1 minute. A final elongation of 5 minutes at 72°C was performed. The PCR products underwent 2% agarose gel electrophoresis stained with 5 *μ*g/ml of red gel with a 100 bp DNA ladder as a molecular weight marker. Migration was performed at a scale of 80 V/cm for 25 min. The amplification bands were visualized and photographed under ultraviolet light.  Table 1 presents the primers of resistance and virulence genes sought.

### 2.5. Data Processing and Analysis

The data were collected and recorded in an Excel 2010 spreadsheet, and the graphs were created using GraphPad Prism 7 software.

## 3. Results

### 3.1. Different Bacterial Species Identified

**Figure 3:**
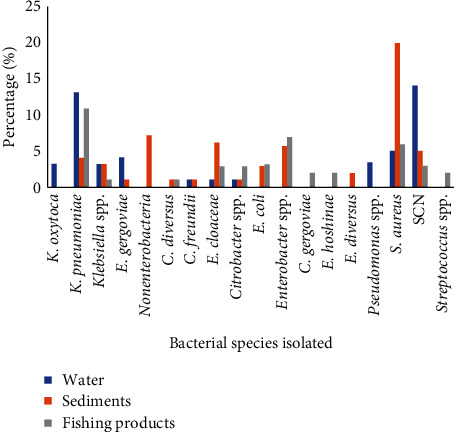
Different bacterial species isolated according to sample type. *K. oxytoca*, *Klebsiella oxytoca*; *K. pneumoniae*, *Klebsiella pneumoniae*; *E. gergoviae*, *Enterobacter gergoviae*; *C. diversus*, *Citrobacter diversus*; *C. freundii*, *Citrobacter freundii*; *E. cloaceae*, *Enterobacter cloaceae*; *E. coli*, *Escherichia coli*; *C. gergoviae*, *Citrobacter gergoviae*; *E. hoshinae*, *Edwarsiella hoshinae*; *E. diversus*, *Enterobacter diversus*; *S. aureus*, *Staphylococcus aureus*; SCN, coagulase-negative staphylococci.

The bacteria identified were mostly enterobacteria followed by Gram-positive cocci. Sediment samples contained 40.97% of the identified bacterial strains followed by water samples (32.63%) and fish product gut samples (26.38%). Nonenterobacterial bacilli were identified in 80% of the sediment samples.  Figure 3 presents the different bacteria isolated according to the type of sample. *Klebsiella pneumoniae* (28.88%) and *Staphylococcus aureus* (58.49%) were the most isolated bacterial strains from all samples collected.

### 3.2. Resistance Profile of Identified Strains

**Figure 4:**
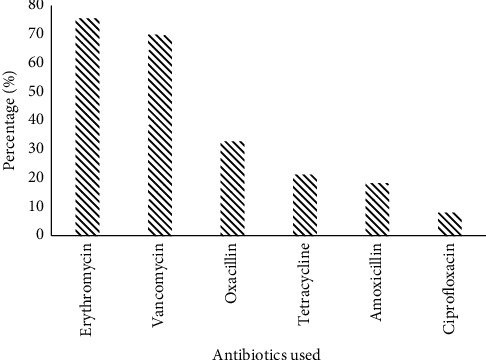
Resistance profile of isolated Gram-positive cocci.

35.84% of the Gram-positive cocci strains identified were multidrug resistant. The greatest resistance was observed to erythromycin (75.47%), vancomycin (69.81%), and oxacillin (32.64%).  Figure 4 shows the resistance profile of Gram-positive cocci.

**Figure 5:**
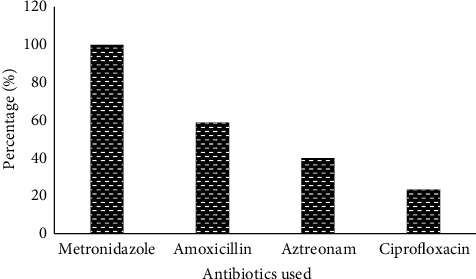
Resistance profile of Gram-negative bacilli.

36.67% of isolated Gram-negative bacilli were multidrug resistant. All strains were resistant to metronidazole (100%). 58.89% resistance was observed to amoxicillin, 40% to aztreonam, and 23.33% to ciprofloxacin.  Figure 5 shows the resistance profile of Gram-negative bacilli.

### 3.3. Molecular Characterization of Isolated Bacteria

The *van A* and *van B* genes were tested only in Gram-positive cocci with resistance to vancomycin. As for the *mecA* gene, it was searched in the genome of all cocci strains and was present in the genome of 25.93% of the strains. The *van B* gene was not found in the genome of any cocci strain unlike *van A* which was present at 3.70% (Table 2).

The resistances genes IMP, VIM, NDM, KPC, OXA-48, OXA-23, *qnrA*, and GES were absent in the genome of all Gram-negative bacilli isolated. The *bla*_TEM_ gene (45.55%) was the most found followed by *bla*_CTX-M-15_ (31.11%) and *bla*_SHV_ (23.33%). Virulence gene *fimH* was found in the genome of 40% of Gram-negative bacilli (Table 3).

## 4. Discussion

The present study was set out to document the level of contamination of the aquatic ecosystem of the Cotonou channel hydrographic complex of Cotonou-Lake Nokoué by antibiotic-resistant bacteria. It should be noted that Lake Nokoué is one of the most important lakes in Benin which allows to satisfy the needs of the populations in fishing products. This study has identified some species of fishery products appreciated and widely consumed by the populations of southern Benin. Among these species, *Sardinella madarensis, Hepsetus odoe, Parachanna obscura, Lutjanus groeensis, Eleotris vittata, Chrysichthys nigrodigitatus, Macrobrachium vollenhovenii, Monodactylus sebae, Clarias gariepinus, Dalophis boulengeri, Tilapia guineensis, Penaeus monodon,* and *Crassostrea tulipa* were found among all the fishermen and resellers of these products. These results corroborate those of Niyonkuru and Lalèyè [27] who found the same families and species in their work relating to the impact of a fishing technique on Lake Nokoué. Sediment samples taken from the lake bottom were the most contaminated with bacteria. This could be explained by sedimentation of bacteria in the lake bottom.

The positive search for pathogenic bacteria in the intestines of fishing products represents a high health risk for the people who consume them. Indeed, the bacterial species most found in the intestines of fish products were *Klebsiella pneumoniae, Enterobacter* spp.*, Staphylococcus aureus*, and *Escherichia coli.* The work of Novotny et al. [28] has also identified pathogenic bacteria found in fish products that could represent a health risk for human populations. Resistance of all strains of Gram-negative bacilli was found. This result could be due to the fact that this antibiotic is considered as a deworming and antispasmodic agent by Beninese populations and taken without control. It should be noted that very few studies have reported the use of metronidazole alone in the treatment of infections induced by Gram-negative bacteria. It would thus be judicious to use it in association with other families of antibiotics and according to the infection to hope for better efficacy [29]. Very high resistances of paramount importance in human health were also noted in both Gram-negative bacteria and isolated Gram-positive cocci. Indeed, 40% of Gram-negative bacteria were resistant to aztreonam, the only monobactam of recourse in case of resistance to carbapenems. Gram-positive cocci were 69.81% resistant to vancomycin. Associated with these resistances, the *bla*_TEM_, *bla*_SHV_, and *bla*_CTX-M-15_ resistance genes were found in the genome of Gram-negative bacilli as well as the *fimH* virulence gene. According to Ruppé et al. [30], the dissemination of plasmid-borne beta-lactamases constitutes by far the most critical resistance problem in Gram-negative bacilli and more specifically in enterobacteria. The dissemination of these genes in the clinic could expose patients to the risk of treatment failure and increased hospitalization time [31]. Several studies have documented the use of extended-spectrum beta-lactamase-producing bacteria [32]. This study is similar to that of Novovic et al. [33] who also isolated Gram-negative bacilli with the *bla*_SHV_ gene from a lake in Bangladesh. The *bla*_CTX-M-15_ gene is the most widely found CTX-M type ESBL in the world. The 31.11% prevalence obtained in this study is consistent with Hawkey's work [34] and Canton and Coque [35]. CTX-M-15 has been found predominantly in *Escherichia coli* but has the ability to rapidly emerge in other Gram-negative bacillus populations [36]. The virulence *fimH* gene gives the bacteria carrying it the ability to adhere to cells and escape the action of antibiotics. Countless studies have documented the presence of this gene in the genome of Gram-negative bacilli, notably in *Escherichia coli*, as in the work of Dadi et al. [37]. In Gram-positive cocci, the *mecA* gene was the most found followed by *van A*, very little found despite the high resistance observed compared to vancomycin. The presence of the *van A* gene induces resistance to glycopeptides. The *mecA* gene indicates the presence of resistance to methicillin [38].

## 5. Conclusion

The data from this study once again raise questions about the appropriate use of antibiotics in agriculture, animal husbandry, and human health in order to prevent multiresistant bacteria from persisting in the environment. The presence of antibiotic-resistant bacteria carrying resistance genes of clinical interest in the aquatic environment is a critical situation that raises fears of an upsurge in infectious diseases that are difficult to treat. This study provides new data on the level of bacteriological contamination of Lake Nokoué in Benin, and given the importance of this aquatic ecosystem, necessary and judicious measures must be taken for an effective depollution of this body of water.

## Figures and Tables

**Table 1:** Primers used for the detection of resistance and virulence genes.

Genes	Primers	Sequence 5′-3′	References
TEM	TEM F	ATGAGTATTCAACATTTCCGC	[16]
	TEM R	CAATGCTTAATCAGTGAGG	

SHV	SHV F	AAGATCCACTATCGCCAGCAG	[16]
	SHV R	ATTCAGTTCCGTTTCCCAGCGG	

CTX-M-15	CTX-M-15F	CACACGTGGAATTTAGGGACT	[16]
	CTX-M-15R	GCCGTCTAAGGCGATAAACA	

IMP	IMP F	GGAATAGAGTGGCTTAATTC	[17]
	IMP R	GGTTTAACAAAACAACCACC	

VIM	VIM F	GCACTTCTCGCGGAGATTG	[18]
	VIM R	CGACGGTGATGCGTACGTT	

GES	GES F	GCAATGTGCTCAACGTTCAAG	[19]
	GES R	GTGCCTGAGTCAATTCTTTCAAAG	

NDM	NDM F	GGCCACACCAGTGACAATATCA	[20]
	NDM R	CAGGCAGCCACCAAAAGC	

KPC	KPC F	GCCGCCAATTTGTTGCTGAA	[21]
	KPC R	GCCGGTCGTGTTTCCCTTT	

OXA-48	OXA-48F	TGTTTTTGGTGGCATCGAT	[22]
	OXA-48R	GTAAMRATGCTTGGTTCGG	

*qnrA*	*qnrA* F	AGGATTTCTCACGCCAGGATT	[23]
	*qnrA* R	CCGCTTTCAATGAAACTGCAA	

OXA-23	OXA-23 F	TTTACTTGCTATGTGGGTTGCT	[24]
	OXA-23 R	ATCACCTGATTATGTCCTTGA	

*van A*	*van A* F	GGGCTGTGAGGTCGGTTG	[25]
	*van A* R	TTCAGTACAATGCGGCCGTTA	

*van B*	*van B* F	TTGTCGGCGAAGTGGATCA	[25]
	*van B* R	AGCCTTTTTCCGGCTCGTT	

*mecA*	*mecA* F	TAATTATCGCAGCAGCTGGTTC	[25]
	*mecA* R	GTTCCCAAACGGAGTATAAGAGTG	

*fimH*	*fimH* F	TACTGCTGATGGGCTGGTC	[26]
	*fimH* R	GGCAATGCTTATTACAGGATGTGC	

**Table 2:** Resistance genes found in the genome of Gram-positive cocci.

Bacterial strains	Resistance genes	
	*mecA*	*van A*
*Staphylococcus aureus*	7/32 (21.88%)	02/32 (6.25%)
SCN	7/21 (33.33%)	0/21 (0.00%)
*Streptococcus* spp.	0/1 (0.00%)	0/1 (0.00%)
**Total**	**14/54 (25.93%)**	**2/54 (3.70%)**

SCN, coagulase-negative staphylococci.

**Table 3:** Resistance and virulence genes found in the genome of Gram-negative bacilli.

Bacterial strains	Resistance genes			Virulence gene
	*bla*_TEM_	*bla*_SHV_	*bla*_CTX-M-15_	*fimH*
*Pseudomonas* spp.	00/02 (0%)	00/02 (0%)	00/02 (0%)	01/02 (50%)
*Klebsiella oxytoca*	03/03 (100%)	02/03 (66.66%)	01/03 (33.33%)	03/03 (100%)
*Klebsiella pneumoniae*	13/28 (46.42%)	7/28 (25%)	12/28 (42.85%)	14/28 (50%)
*Klebsiella* spp.	04/07 (57.14%)	02/07 (28.57%)	02/07 (28.57%)	03/07 (42.85%)
*Enterobacter gergoviae*	03/05 (60%)	03/05 (60%)	01/05 (20%)	03/05 (60%)
*Enterobacter cloaceae*	04/10 (40%)	01/10 (10%)	02/10 (20%)	03/10 (30%)
*Enterobacter diversus*	00/01 (0%)	00/01 (0%)	00/01 (0%)	01/01 (100%)
*Enterobacter* spp.	08/12 (66.66%)	03/12 (25%)	07/12 (58.33%)	04/12 (33.33%)
*Citrobacter diversus*	00/01 (0%)	00/01 (0%)	00/01 (0%)	01/01 (100%)
*Citrobacter freundii*	01/02 (50%)	00/02 (0%)	01/02 (50%)	01/02 (50%)
*Citrobacter gergoviae*	01/01 (100%)	01/01 (100%)	00/01 (0%)	01/01 (100%)
*Citrobacter* spp.	00/04 (0%)	01/04 (25%)	00/04 (0%)	00/04 (0%)
*Escherichia coli*	01/05 (20%)	02/05 (40%)	01/05 (20%)	01/05 (20%)
*Edwarsiella hoshinae*	00/01 (0%)	00/01 (0%)	00/01 (0%)	00/01 (0%)
Nonenterobacteria	03/08 (37.5%)	01/08 (12.5%)	01/08 (12.5%)	00/08 (0%)
**Total**	**41/90**	**21/90**	**28/90**	**36/90**

## Data Availability

The data used to support this study are included within the article.
